# Supplementary material for: Predicting T‐cell quality during manufacturing through an artificial intelligence‐based integrative multiomics analytical platform
Source: Bioeng Transl Med. 2022 Jan 4;7(2):e10282. doi: 10.1002/btm2.10282 (PMC9115702; doi:10.1002/btm2.10282)
Supplement: Supplementary file 1 — Appendix S1: Supporting Information [file BTM2-7-e10282-s002.docx]

Predicting T Cell Quality During Manufacturing Through an Artificial Intelligence-based Integrative Multi-Omics Analytical Platform

Valerie Y. Odeh-Couvertier^1,6^, Nathan J. Dwarshuis^2,6^, Maxwell B. Colonna^3,6^, Bruce L. Levine^4^, Arthur S. Edison^3^, Theresa Kotanchek^5^, Krishnendu Roy^2^, and Wandaliz Torres-Garcia^1,7,*^

^1^Department of Industrial Engineering, University of Puerto Rico Mayagüez, Mayagüez, PR, 00681, USA

^2^The Wallace H. Coulter Department of Biomedical Engineering, Georgia Institute of Technology, Atlanta, GA, 30318, USA

^3^Departments of Genetics and Biochemistry & Molecular Biology, Complex Carbohydrate Research Center, University of Georgia, Athens, GA, 30602, USA

^4^Center for Cellular Immunotherapies, Perelman School of Medicine, University of Pennsylvania, Philadelphia, PA, 19104, USA

^5^Evolved Analytics LLC, Rancho Santa Fe, CA, USA

^6^These authors contributed equally.

^7^Lead Contact

*Correspondence: [wandaliz.torres@upr.edu](mailto:wandaliz.torres@upr.edu)

**Supplemental information**

Dataset S1 (separate file: btm_SuppDatasetS1.xlsx ). Process parameters, Cytokine, NMR metabolomics, end-product responses (i.e., T_N_+T_CM_ cells), other cell morphology details can be found for both experiments performed (DOE, ADOE). Column names are self-explanatory, and their categories followed as Experiments information, Process Parameters, Media cytokine secretion at day 6, 8, 11, and 14, Media NMR analysis at day 4, 6, 8, 11, and 14, and other info.

***Supplementary Figure***


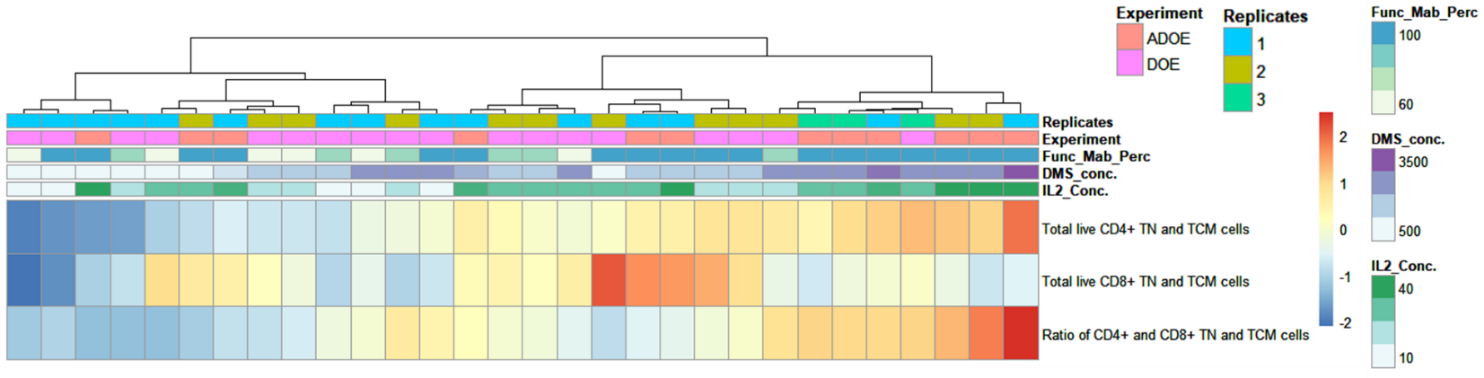


**Supp.Fig.S1.** **Heatmap display of hierarchical clustering for all three T_N_+T_CM_ endpoint responses** using Ward.D agglomeration and Euclidean distance. Replicates represent the number of samples for that particular process parameter combination.


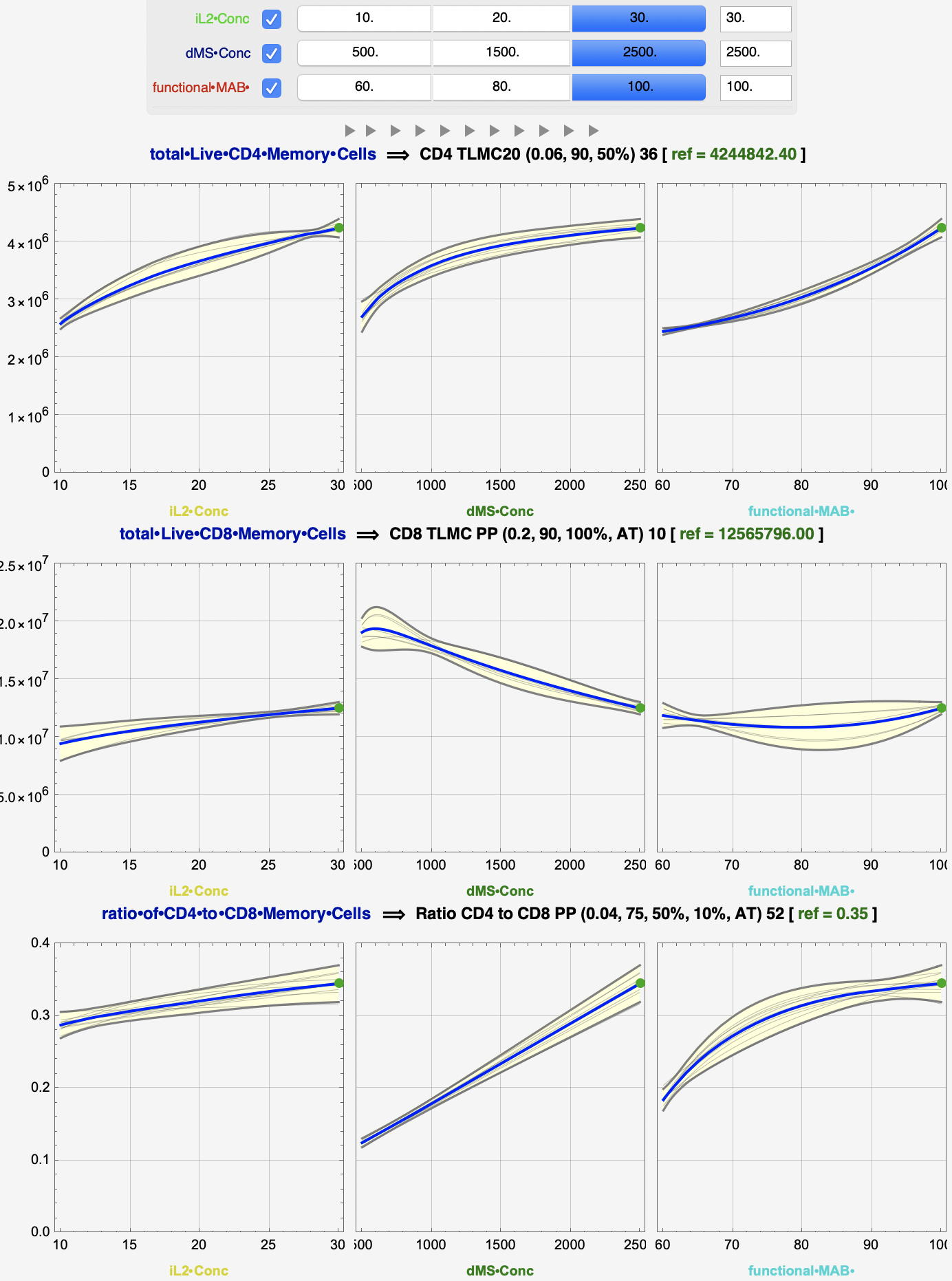


**c**

**b**

**a**

**Supp.Fig.S2. Symbolic regression ensemble plots as given by DataModeler optimizing for** **Total live CD4^+^ T_N_+T_CM_ cells.** Predicted response profiles of a) Total live CD4^+^ T_N_+T_CM_ cells, b) Total live CD8^+^ T_N_+T_CM_ cells and c) Ratio of CD4^+^ to CD8^+^ T_N_+T_CM_ cells at the predicted optimum for Total live CD4^+^ T_N_+T_CM_ cells.


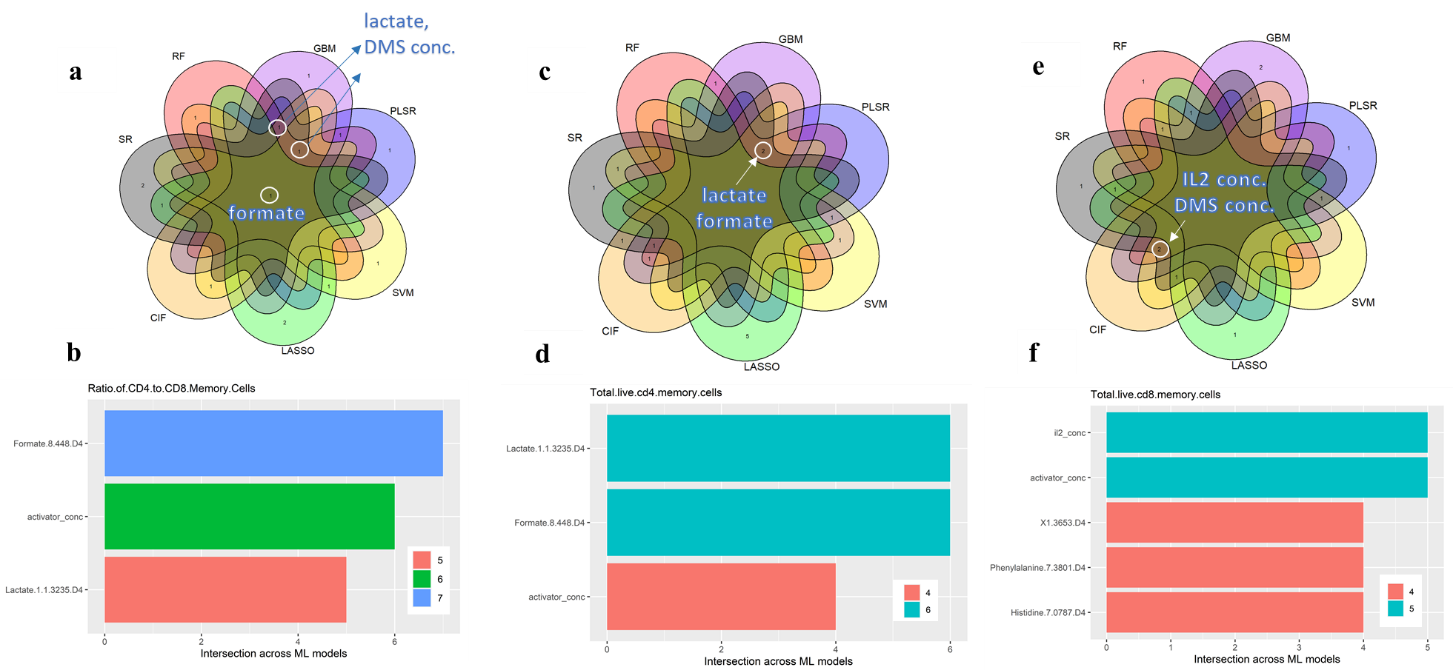


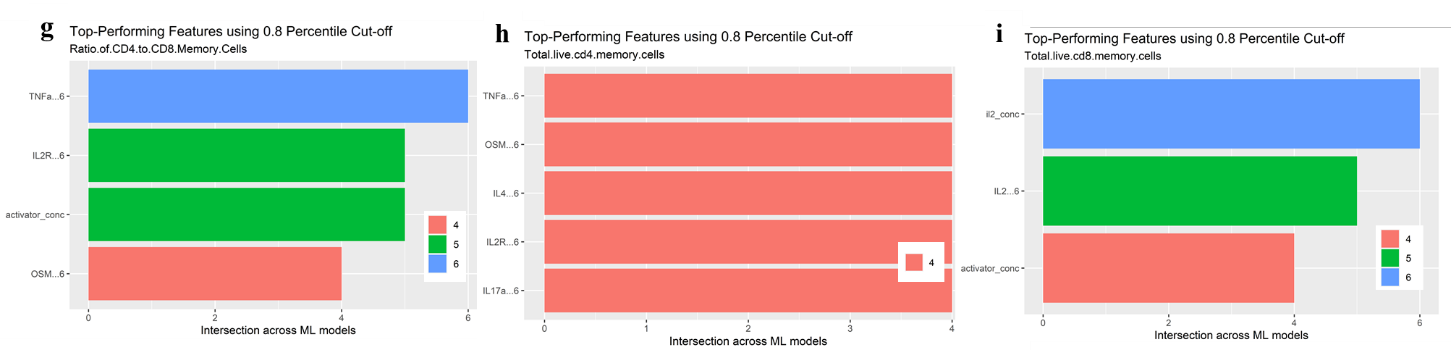


**Supp.Fig.S3.** **Overall feature consensus analysis of top-performing features in single-omics (a-f) NMR models at day 4 and (g-i) Cytokine models at day 6** for **a,b,g**) ratio of total live CD4^+^ to CD8^+^ T_N_+T_CM_ cells, **c,d,h**) total live CD4^+^ T_N_+T_CM_ cells, and **e,f,i**) total live CD8^+^ T_N_+T_CM_ cells


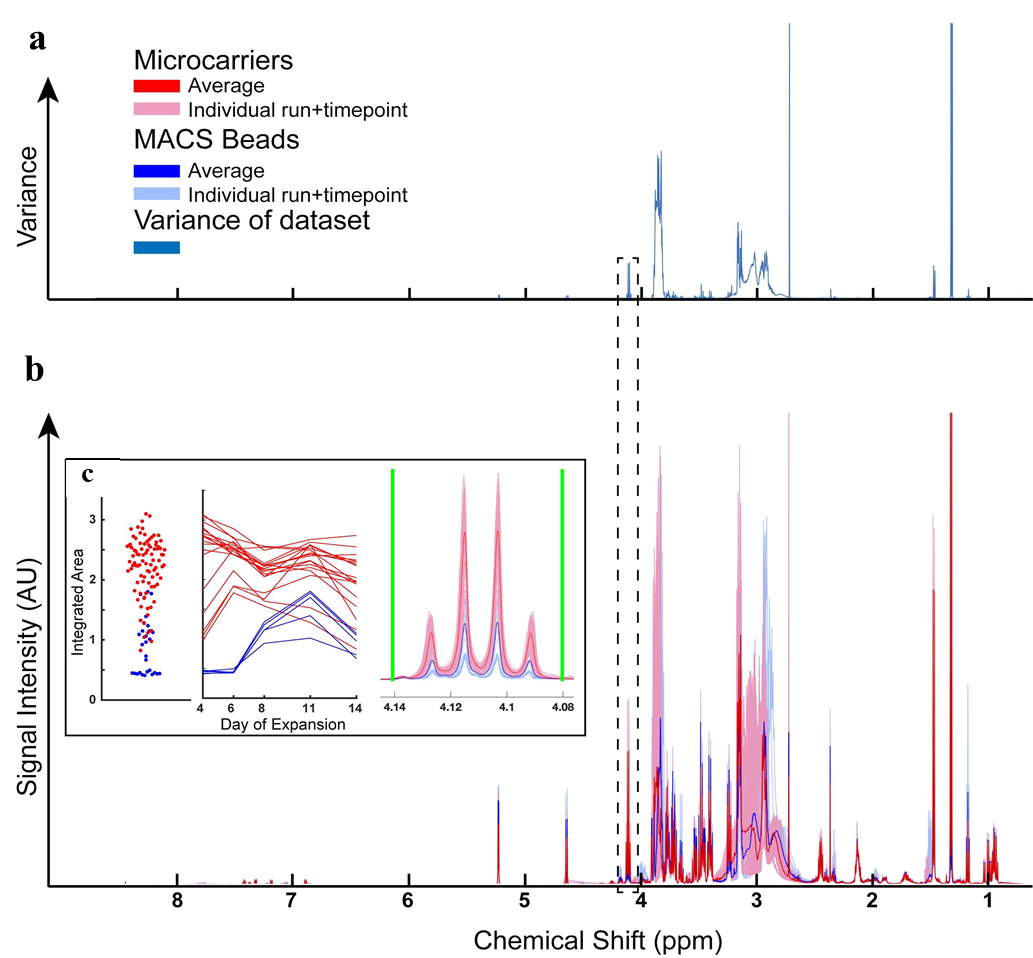


**Supp.Fig.S4.** **Variance based feature selection of NMR features for computational modeling.** a) Plot of variance across ^1^H NMR spectrum for all experimental samples. X-axis matched to below. b) Plot of all experimental spectra for both microcarrier and MACS bead process runs. Averages shown in bold lines. c) Integration of feature highlighted in dashed box. Far right plot shows overlay of all experimental spectra and averages for both groups. Vertical green lines correspond to boundaries for feature integration. Center plot shows the trajectory of integrated values for individual runs (represented as continuous lines) over the expansion period indicated on X-axis. Far left-plot shows a distribution of integrated values for all samples over all timepoints.

**A**
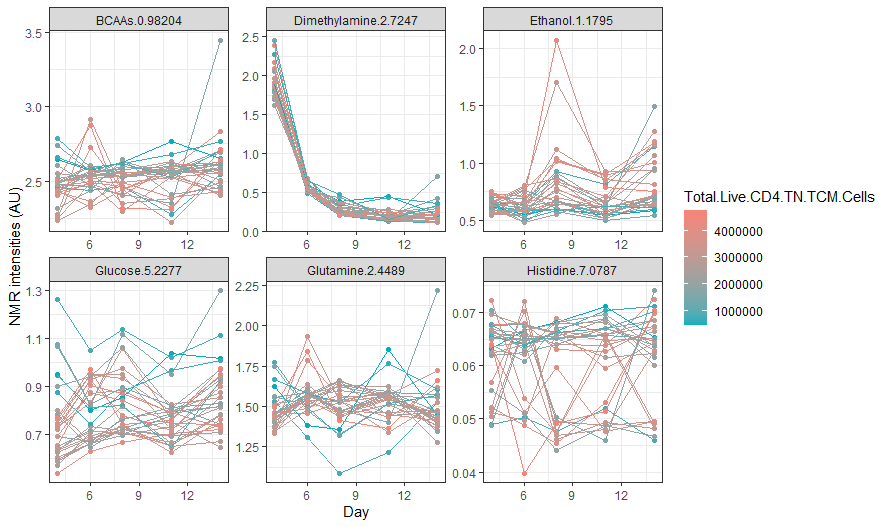


**B
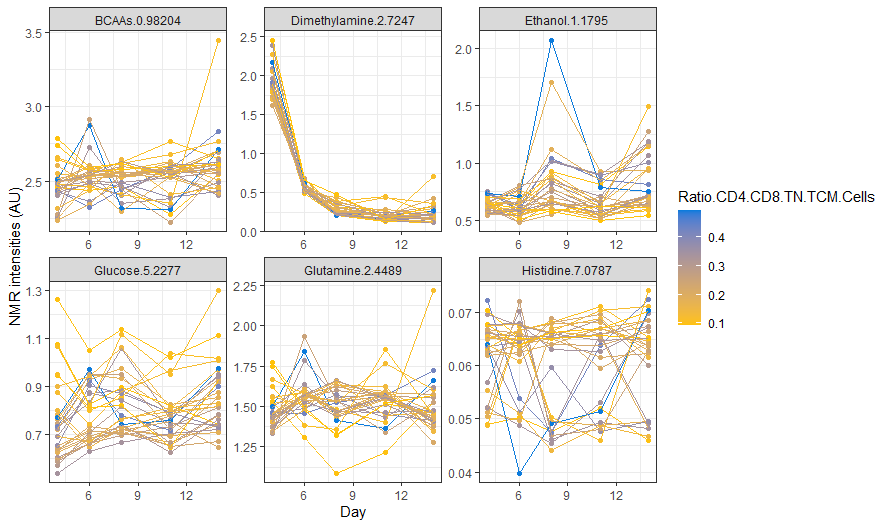
**

**C**
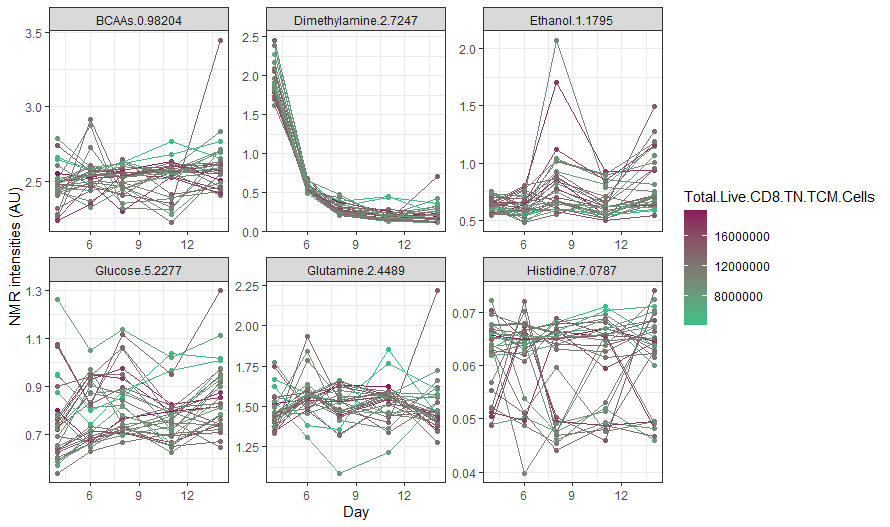


**Supp.Fig.S5. Media NMR intensities across monitoring times** for a) total live CD4^+^ T_N_+T_CM_ cells, b) ratio CD4^+^/CD8^+^ T_N_+T_CM_, and c) total live CD8^+^ T_N_+T_CM_ cells.

**a**
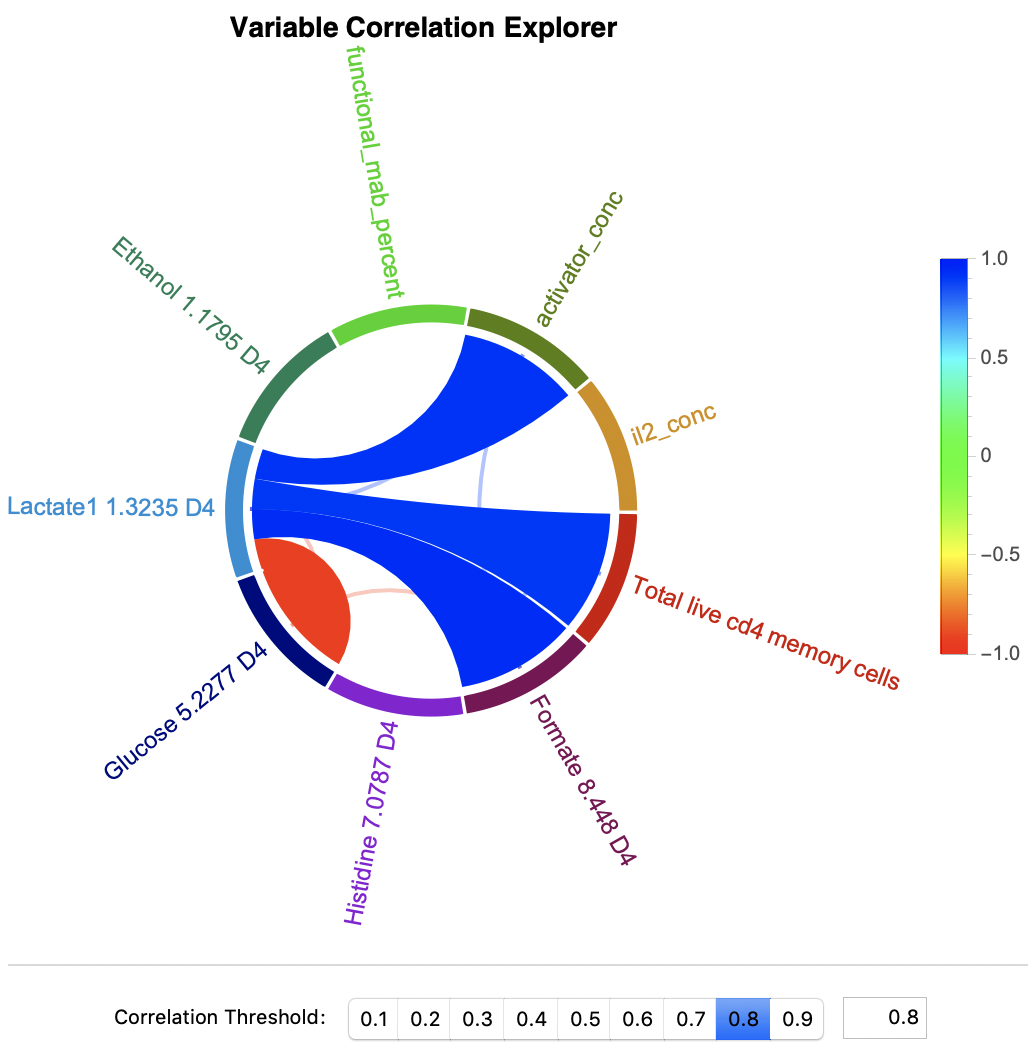
**b**
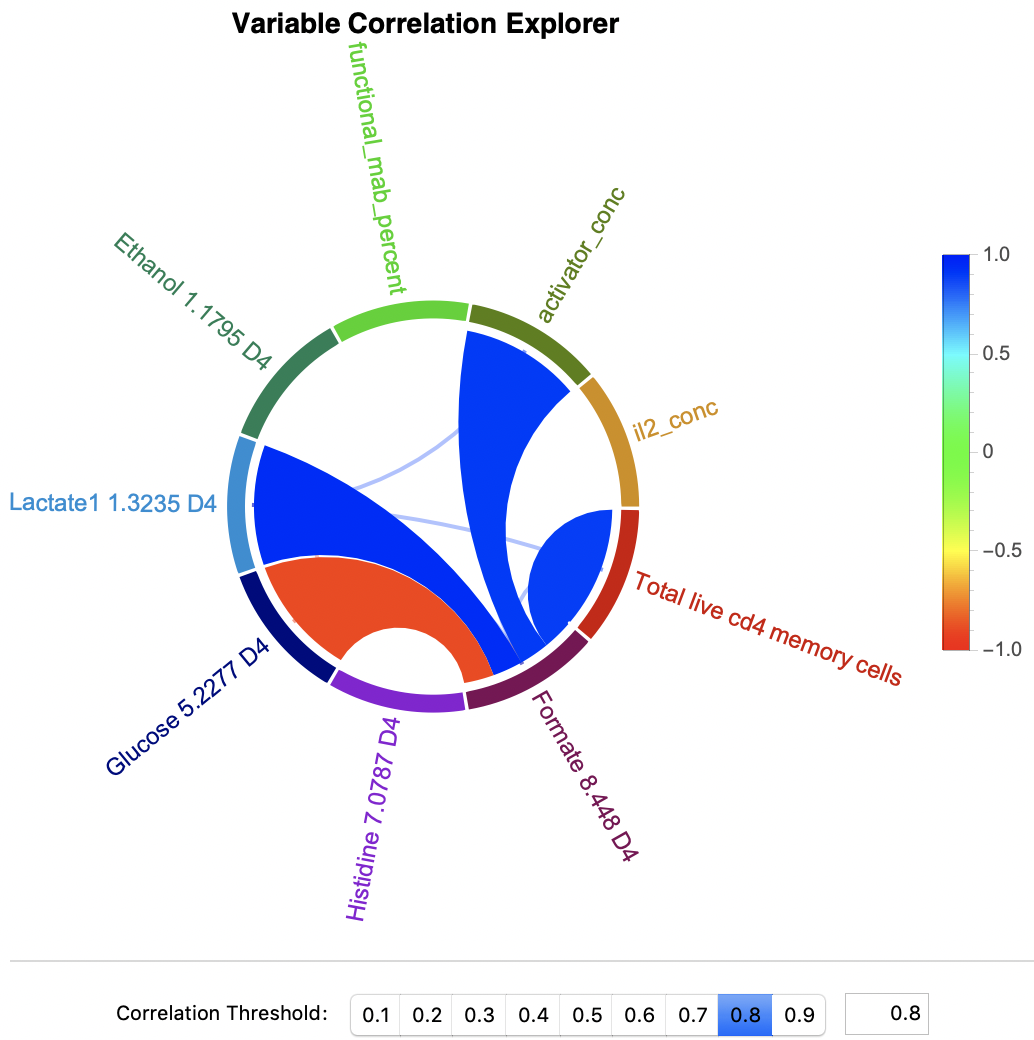


**c**
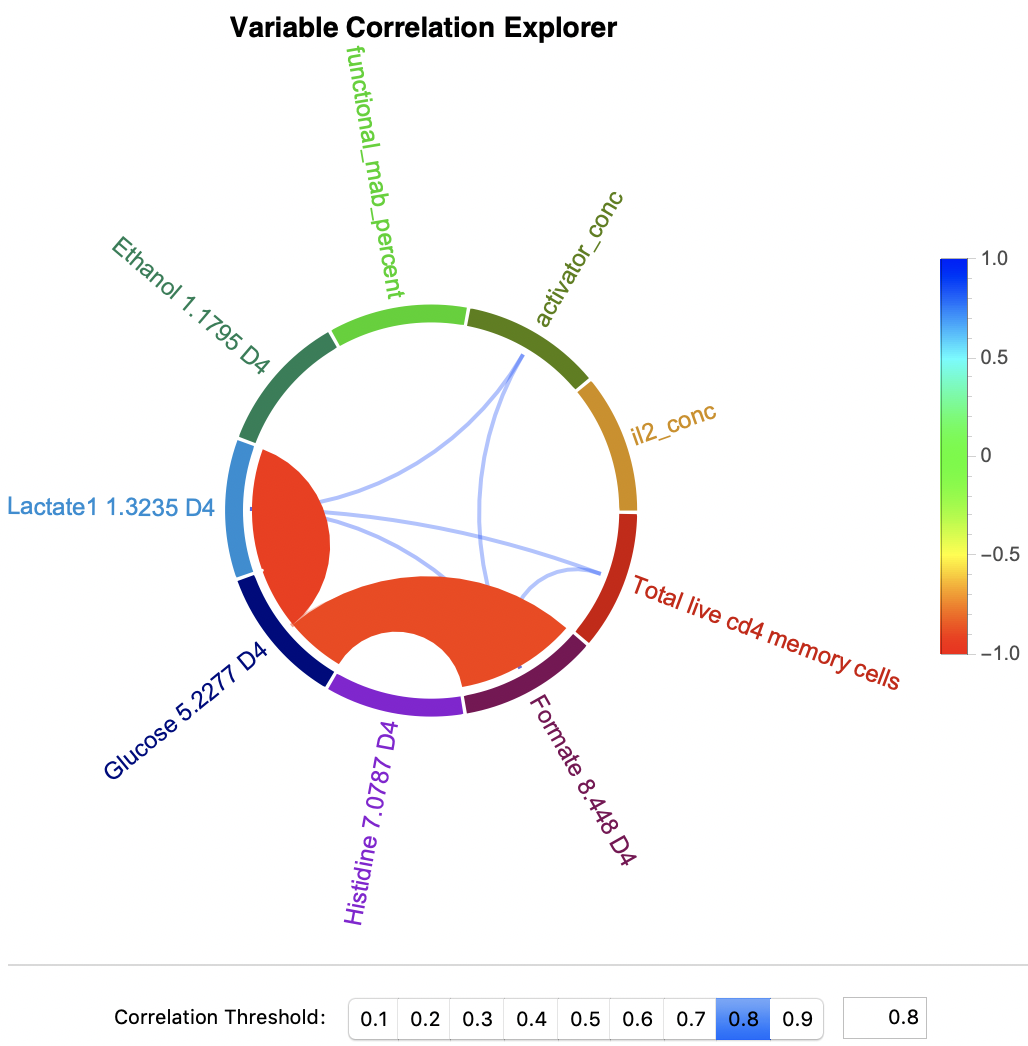
**d**
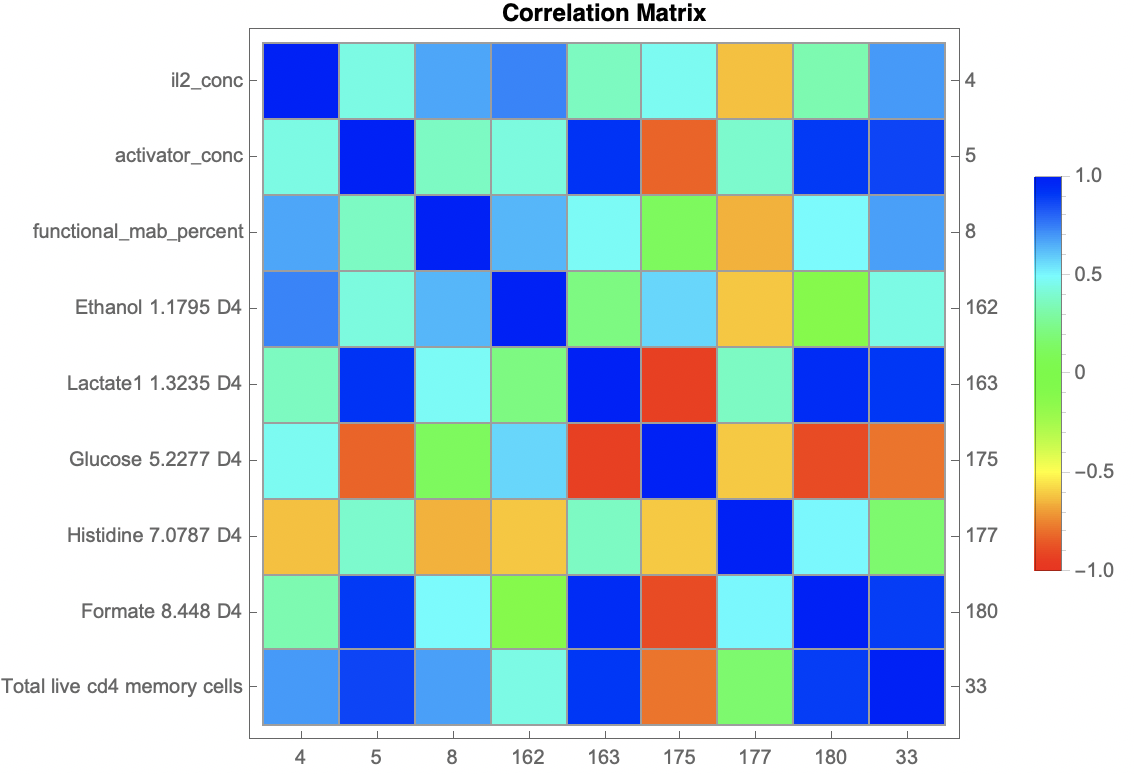


**Supp.Fig.S6: NMR Feature Correlation for SR-DataModeler models for NMR media analysis at day 4**: **a)** lactate is strongly positively correlated with formate, DMS Conc and Total Live CD4^+^ and negatively correlated with glucose; **b)** formate is strongly positively correlated with lactate, DMS Conc and Total Live CD4^+^ and negatively correlated with glucose; **c)** glucose is negatively correlated with lactate and formate; **d)** NMR correlation Matrix.

**
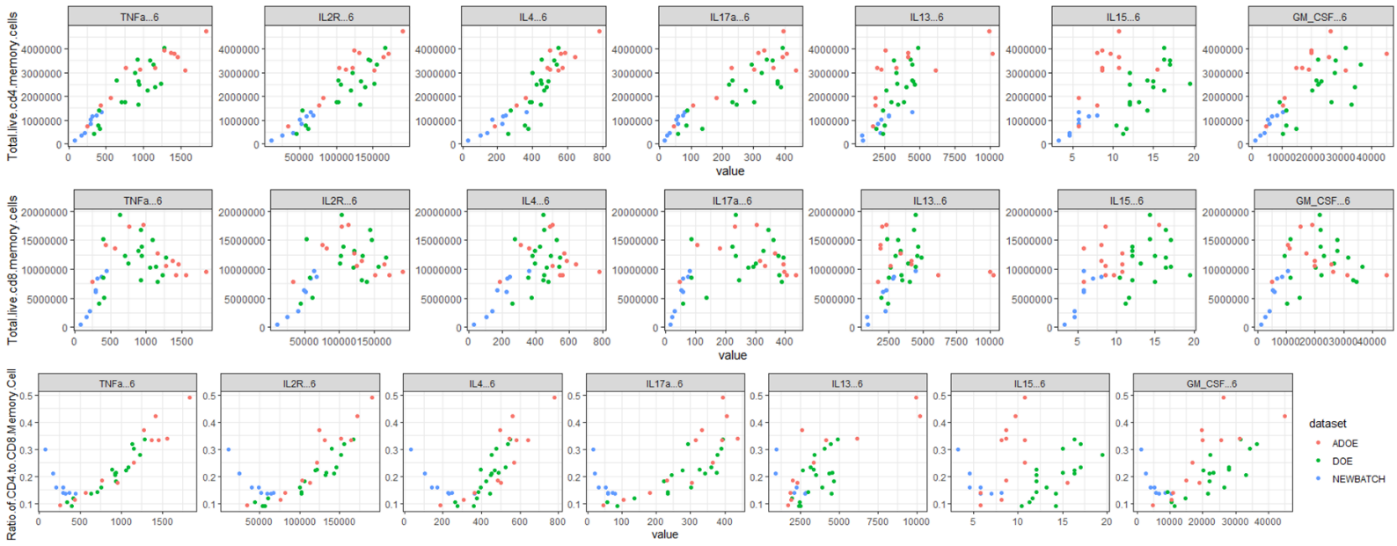
 Supp.Fig.S7:** Intensity values for predictive cytokine features from media analysis on T cell culturing at day 6 for T_N_ + T_CM_ responses.

**
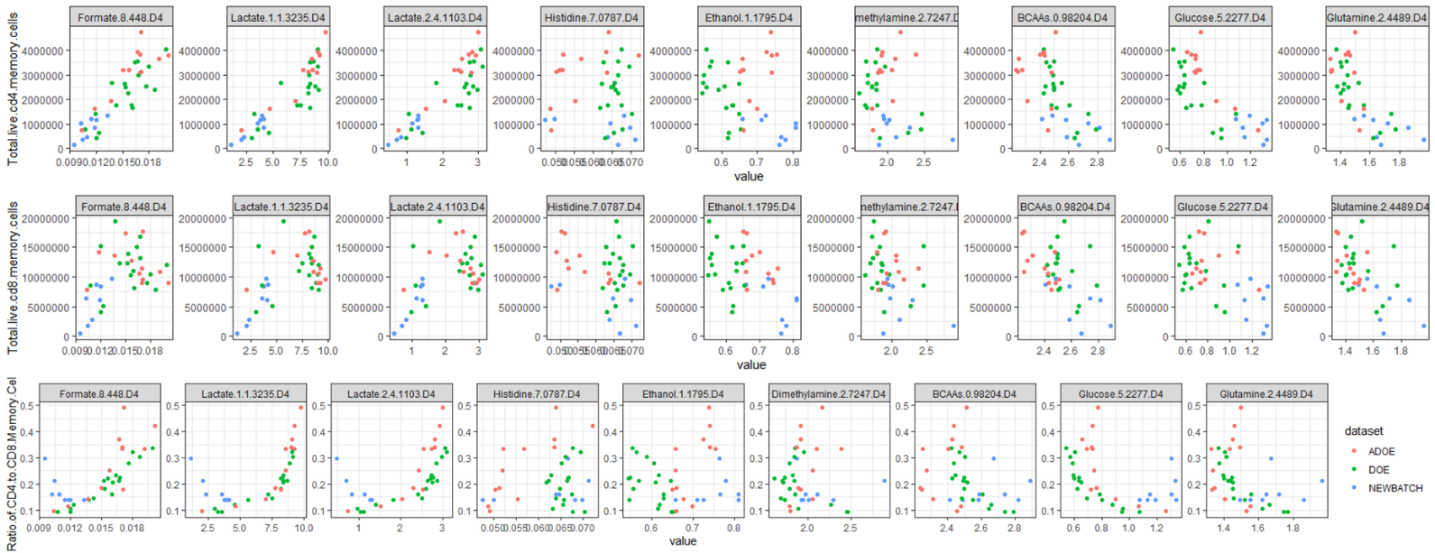
Supp.Fig.S8:** Intensity values for predictive NMR features from media analysis on T cell culturing at day 4 for T_N_ + T_CM_ responses.

***Supplementary Tables***

**Supp.Table.S1.** Summary Statistics for Day 14 Total Live (CD4+, CD8+) T_N_ and T_CM_ cells and Ratios for DOE/ADOE

| **Response** | **Experiment** | **Minimum** | **Median** | **Mean** | **Maximum** |
| --- | --- | --- | --- | --- | --- |
| Total live CD4+ T_N_ and T_CM_ cells | DOE: 18-runs | 4.3 x 10^5^ | 2.5 x 10^6^ | 2.3 x 10^6^ | 4.0 x 10^6^ |
|  | ADOE: 12-runs | 7.4 x 10^5^ | 3.2 x 10^6^ | 3.1 x 10^6^ | 4.7 x 10^6^ |
| Total live CD8+ T_N_ and T_CM_ cells | DOE: 18-runs | 4.1 x 10^6^ | 1.2 x 10^7^ | 1.1 x 10^7^ | 1.9 x 10^7^ |
|  | ADOE: 12-runs | 7.8 x 10^6^ | 1.1 x 10^6^ | 1.2 x 10^6^ | 1.8 x 10^7^ |
| Ratio live CD4+/CD8+ T_N_ and T_CM_ cells | DOE: 18-runs | 0.09 | 0.21 | 0.20 | 0.34 |
|  | ADOE: 12-runs | 0.09 | 0.29 | 0.27 | 0.49 |

**Supp.Table.S2.** Variable combinations across responses from top-performing SR DataModeler

| **Response** | **Predictors** | **Top Symbolic Regression DataModeler Combinations** |
| --- | --- | --- |
| **Ratio CD4+/CD8+ T_N_+T_CM_ cells** | **PP+N4** | DMS Conc + Functional Mabs %+Histidine+Formate+Lactate |
|  |  | IL2 Conc+DMS Conc + Functional Mabs %+Histidine+Formate+Ethanol |
|  |  | IL2 Conc+DMS Conc + Functional Mabs %+Histidine+Lactate |
|  | **PP+N6** | DMS Conc + Functional Mabs%+UK 1.3653+ Dimethylamine+ Glycine+UK 7.5387 |
|  |  | DMS Conc+Functional Mabs%+ Lactate1+Histidine +UK 7.5387 |
|  | **PP+S6** | DMS Conc + Functional Mabs %+GMCSF+IL2R+MIF |
|  |  | DMS Conc +GMCSF+IL2R+ IL5+MIF |
|  |  | DMS Conc + Functional Mabs %+GMCSF+TNFa |
|  | **PP+S6+N6** | GMCSF+ IL3+TNFa+ Tyrosine+Formate |
|  |  | IL3+TNFa+ Tyrosine+Formate |
|  |  | IL3+TNFa+Formate+UK 7.5387 |
| **Total Live CD4+ T_N_+T_CM_ cells** | **PP+N4** | IL2 Conc+DMS Conc+Functional Mabs%+Ethanol+Lactate |
|  |  | IL2 Conc+DMS Conc+Functional Mabs%+Ethanol+Formate |
|  |  | IL2 Conc+DMS Conc+Functional Mabs%+Ethanol+Lactate+Formate |
|  |  | IL2 Conc+DMS Conc+Functional Mabs%+Ethanol+Formate+Histidine |
|  |  | IL2 Conc+Functional Mabs%+Ethanol+Dimethylamine+Lactate |
|  | **PP+N6** | IL2 Conc + DMS Conc + Functional Mabs% + Lactate + Phenylalanine |
|  |  | IL2 Conc + DMS Conc + Functional Mabs%+UK 4.1784 |
|  | **PP+S6** | IL2 Conc + IL13 + IL15 + IL17a + IL2R |
|  |  | IL2 Conc + IFN alpha+ IL13 + IL15 + IL2R |
|  |  | IL2 Conc + IL13 + IL15 + IL2R |
|  | **PP+S6+N6** | IL2 Conc+IFN Alpha+ IL13+IL15+Histidine |
|  |  | IL2 Conc+ IL13+IL15+IL17a+IL2R+Glycine |
|  |  | IL2 Conc+IL13+IL15+IL2R+MIF+Glycine |
| **Total Live CD8+ T_N_+T_CM_ cells** | **PP +N4** | IL2 Conc + DMS Conc + Lactate + Ethanol + Histidine + BCAAs |
|  |  | IL2 Conc + DMS Conc + Functional Mabs% + Lactate + Ethanol + Histidine |
|  |  | IL2 Conc + DMS Conc + Formate + Ethanol + Glucose + BCAAs |
|  |  | IL2 Conc + DMS Conc + Formate + Glucose + BCAAs |
|  |  | IL2 Conc + DMS Conc + Ethanol + Lactate+ Glutamine |
|  | **PP+N6** | IL2 Conc + DMS Conc + Ethanol + Pyruvate + UK 7.5387 |
|  |  | IL2 Conc + DMS Conc + Ethanol +Tyrosine |
|  |  | IL2 Conc + DMS Conc + Ethanol +Lactate |
|  | **PP+S6** | IL2 Conc+ DMS Conc+ IL15+IL17a+TNFa |
|  | **PP+S6+N6** | IL2 Conc +DMS Conc + GM CSF+IL15+IL17a+UK 1.5208 |
|  |  | IL2 Conc +DMS Conc + GM CSF+IL15+IL17a+IL2R |
|  |  | IL2 Conc +DMS Conc +IL15+IL17a+TNFa |

Note: UK means unknown or unidentified.

**Supp.Table.S3.** Variables present in >30% of the top-performing Symbolic Regression models from DataModeler (R2≥ 90%, Complexity ≤ 100) for the different end-product responses.

| **Input** | **Ratio CD4+/CD8+ T_N_+T_CM_ cells** | **Total Live CD4+ T_N_+T_CM_ cells** | **Total Live CD8+ T_N_+T_CM_ cells** |
| --- | --- | --- | --- |
| **PP+N4** | DMS Conc  Functional Mabs %  Lactate  Formate  Histidine  Ethanol | IL2 Conc  DMS Conc  Functional Mab %  Lactate  Formate  Ethanol | IL2 Conc  DMS Conc  Lactate  Formate  Histidine  BCAAs  Ethanol |
| **PP+N6** | Functional Mabs%  DMS conc  UK 75387  Dimethylamine  Glycine  UK 13653  Lactate  Histidine | IL2 Conc  Functional Mab %  DMS Conc  Lactate  Phenylalanine  UK41784 | IL2 Conc  DMS Conc  Ethanol  UK 75387  Tyrosine  Pyruvate  Lactate |
| **PP+S6** | DMS conc  GMCSF  IL2R  MIF  IL5  Functional Mabs %  TNFa | IL Conc  IL2R  IL13  IL15  IL17a  IFN alpha  MIF | IL2 Conc  DMS Conc  IL15  IL17a  TNFa |
| **PP+S6+N6** | TNFa  IL3  Formate  Tyrosine  GMCSF  UK75387 | IL2 Conc  IL2R  IL13  IL15  Glycine  Histidine  IL17a  MIF  IFN alpha | IL2 Conc  IL15  DMS Conc  IL17a  GM CSF  UK 15208  IL2R  TNFa |

***Supplementary Materials and Methods***

**Overall multi-omics study design and development.** The first DOE resulted in a randomized 18-run I-optimal custom design where each DMS parameter was evaluated at three levels: IL2 concentration (10, 20, and 30 U/μL), DMS concentration (500, 1500, 2500 carrier/μL), and functionalized antibody percent (60%, 80%, 100%). These 18 runs consisted of 14 unique parameter combinations where 4 of them were replicated twice to assess prediction error. Process parameters for the ADOE were evaluated at multiple levels: IL2 concentration (30, 35, and 40 U/μL), DMS concentration (500, 1000, 1500, 2000, 2500, 3000, 3500 carrier/μL), and functionalized antibody percent (100%) as depicted in Fig.1*B*. To further optimize the initial region explored (DOE) in terms of total live CD4^+^ T_N_+T_CM_ cells, a sequential adaptive design-of-experiment (ADOE) was designed with 10 unique parameter combinations, two of these replicated twice for a total of 12 additional samples (Fig.1*B*). The fusion of cytokine and NMR profiles from media to model these responses included 30 cytokines from a custom Thermo Fisher ProcartaPlex Luminex kit and 20 NMR features. These 20 spectral features from NMR media analysis were selected out of approximately 250 peaks through the implementation of a variance-based feature selection approach and some manual inspection steps.

**Microcarrier fabrication.** Degradable microscaffolds were fabricated as previously described^1^. Briefly, gelatin microcarriers (CuS, GE Healthcare DG-2001-OO) were suspended at 20 mg*/*mL in 1X phosphate-buffered saline (PBS). Sulfo-NHS-biotin (SNB) (Thermo Fisher 21217 or Apex Bio A8001) was dissolved at 10 µM in ultrapure water and 7*.*5 µL SNB*/*mL PBS was added to carrier suspension and allowed to react for 60 min. After washing the carriers three times in PBS, 40 µg*/*mL streptavidin (Jackson Immunoresearch 016-000-114) was added and allowed to react for 60 min. Biotinylated mAbs against human CD3 and CD28 were combined in a 1:1 mass ratio and added to the carriers at 2 µg mAbs*/*mg carriers. To vary the surface concentration of the antibodies, the anti-CD3/anti-CD28 mAb mixture was further combined with a biotinylated isotype control to reduce the overall fraction of targeted mAbs. mAbs were allowed to bind to the carriers for 60 min. All mAbs were low endotoxin azide-free (Biolegend custom, LEAF specification). Fully functionalized DMSs were washed in sterile PBS and washed once again in the cell culture media to be used for the T cell expansion. The surface concentration of the antibodies was quantified as previously described using a bicinchoninic acid assay (BCA) kit (Thermo Fisher 23227)^1^.

**Flow cytometry.** At the end of culture, at least 1e5 T cells from each run were washed with PBS once, resuspended in PBS, and stained with Zombie UV (Biolegend, 423107) for 30 minutes at room temperature in the dark at a 1:1000 dilution. Cells were spun and resuspended in FACS buffer (1X PBS, 2% bovine serum albumin, 5 mM EDTA) and were stained with antibodies according to **Supp.MM.Table.1** for 60 minutes in the dark at 4C.

**Supp.MM.Table.1. Flow cytometry antibodies**

| **Antigen** | **Fluorophore** | **Vendor** | **Cat Number** |
| --- | --- | --- | --- |
| CD3 | APC-Fire | Biolegend | 34839 |
| CD4 | PerCP-Cy5.5 | BD | 561438 |
| CCR7 | AF647 | BD | 561438 |
| CD62L | PE | BD | 341012 |

**Cytokine measurements.** Cytokines were measured using a custom ProcartaPlex Luminex kit (Thermo Fisher). The assay was performed using media samples taken at various time points throughout the T cell culture according to the manufacturer's instructions with modifications to half the reagent requirements. Briefly, an 8-point standard curve was created with all included standards. 25 μL magnetic beads were added to all required wells and washed three times. 25 μL of each standard or sample was added to the wells and the plate was sealed and spun at 850 rpm for 120 minutes followed by three washes. 12.5 μL detection antibody was added followed by sealing the plate and spinning for 60 minutes at 850 rpm and three washes. 25 μL streptavidin PE was added followed by the same spin and wash steps. 120 μL of reading buffer was added to the plate, the plate was analyzed on a BioPlex 200 (BioRad). Any samples that were majority over-range (denoted as “OOR >” in the output spreadsheet) were deemed too concentrated at run at 1/10th their original concentration to put them within range. All samples were run without technical replicates. Luminex data was preprocessed using R for inclusion in the analysis pipeline as follows. Any cytokine level that was over-range (“OOR >” in output) was set to the maximum value of the standard curve for that cytokine. Any value that was under-range (“OOR <” in output spreadsheet) was set to zero. All values that were extrapolated from the standard curve were left unchanged. Data available at **Supp.Dataset.1**.

**NMR unknown identification.** Several low abundance features selected for analysis did not have database matches and were not annotated. Statistical total correlation spectroscopy^2^ suggested that some of these unknown features belonged to the same molecules (not shown). Additional multidimensional NMR experiments will be required to determine their identity.

**Symbolic regression.** Symbolic regression (SR) was done using Evolved Analytics’ Data Modeler software (Evolved Analytics LLC, Midland, MI). Data Modeler utilizes genetic programming to evolve symbolic regression models (both linear and non-linear) rewarding simplicity and accuracy. Using the selection criteria of highest accuracy (R^2^>90% or noise-power) and lowest complexity, the top-performing models were identified. Driving variables, variable combinations, and model dimensionality tables were generated. The top-performing variable combinations were used to generate model ensembles. In this analysis, Data Modeler’s *SymbolicRegression* function was used to develop explicit algebraic (linear and nonlinear) models. The fittest models were analyzed to identify the dominant variables using the *VariablePresence* function, the dominant variable combinations using the *VariableCombinations* function, and the model dimensionality (number of unique variables) using the *ModelDimensionality* function. *CreateModelEnsemble* was used to define trustable model ensembles using selected variable combinations and these were summarized (model expressions, model phenotype, model tree plot, ensemble quality, model quality, variable presence map, ANOVA tables, model prediction plot, exportable model forms) using the *ModelSummaryTable* function. Ensemble prediction and residual performance were respectively assessed via the *EnsemblePredictionPlot* and *EnsembleResidualPlot* subroutines. Model maxima (*ModelMaximum* function) and model minima (*ModelMinimum* function) were calculated and displayed using the *ResponsePlotExplorer* function. Trade-off performance of multiple responses was explored using the *MultiTargetResponseExplorer* and *ResponseComparisonExplorer* with additional insights derived from the *ResponseContourPlotExplorer*. Graphics and tables were generated by Data Modeler. These model ensembles were used to identify predicted response values, potential optima in the responses, and regions of parameter values where the predictions diverge the most.

**Other ML Methods.** Non-parametric tree-based ensembles were done through the *randomForest, gbm,* and *cforest* regression functions in R, for random forest, gradient boosted trees, and conditional inference forest models, respectively. Both random forest and conditional inference forest construct multiple decision trees in parallel, by randomly choosing a subset of features at each decision tree split, in the training stage. Random forest individual decision trees are split using the Gini Index, while conditional inference forest uses a statistical significance test procedure to select the variables at each split, reducing correlation bias. In contrast, gradient boosted trees construct regression trees in series through an iterative procedure that adapts over the training set. This model learns from the mistakes of previous regression trees in an iterative fashion to correct errors from its precursors' trees (i.e., minimize mean squared errors). Prediction performance was evaluated using leave-one-out cross-validation (LOO)-R^2^ and permutation-based variable importance scores assessing % increase of mean squared errors (MSE), relative influence based on the increase of prediction error, coefficient values for RF, GBM, and CID, respectively. Partial least squares regression was executed using the *plsr* function from the *pls* package in R while LASSO regression was performed using the *cv.glmnet* R package, both using leave-one-out cross-validation. Finally, the *kernlab* R package was used to construct the Support Vector Machine regression models.

Parameter tuning was done for all models in a grid search manner using the *train* function from the *caret* R package using LOO-R^2^ as the optimization criteria. Specifically, the number of features randomly sampled as candidates at each split (mtry) and the number of trees to grow (ntree) were tuned parameters for random forest and conditional inference forest. In particular, minimum sum of weights in a node to be considered for splitting and the minimum sum of weights in a terminal node were manually tuned for building the CIF models. Moreover, GBM parameters such as the number of trees to grow, maximum depth of each tree, learning rate, and the minimal number of observations at the terminal node, were tuned for optimum LOO-R^2^ performance as well. For PLSR, the optimal number of components to be used in the model was assessed based on the standard error of the cross-validation residuals using the function *selectNcomp* from the *pls* package. Moreover, LASSO regression was performed using the *cv.glmnet* package with *alpha* = 1. The best lambda for each response was chosen using the minimum error criteria. Lastly, a fixed linear kernel (i.e., svmLinear) was used to build the SVM regression models evaluating the cost parameter value with best LOO-R^2^. Prediction performance was measured for all models using the final model with LOO-R^2^ tuned parameters. **Supp.MM.Table.2** shows the parameter values evaluated per model at the final stages of results reporting. Machine learning implementation codes used in this work are available at GitHub (<https://github.com/wandaliz/CMaT_TCell_MachineLearning/>). DataModeler information can be requested at <http://www.evolved-analytics.com/>.

**Supp.MM.Table.2.** ML parameter values evaluated and tuned

| **ML Model** | **Tuned Parameter Values** |
| --- | --- |
| RF | ntree=c(500,1000,1500,2000,2500)  mtry=all possibilities |
| GBM | interaction.depth=c(1:4)  n.trees = (1:20)*10  shrinkage=c(0.1,0.01, 0.02)  n.minobsinnode=c(2:6)  bag.fraction=0.5 |
| CIF | mtry=all possibilities  ntree*=100  minsplit* = 6  minbucket* = 3 |
| LASSO | alpha=1  lambda=seq(0.001,0.05,by = 0.001) |
| PLSR | ncomp = 1:15 |
| SVM | svmLinear  cost=seq(0.05,2,.05) |
|  | *other values besides the ones shown were optimized manually |

**Machine Learning Consensus Analysis.** All regression methods were executed, and the high-performing models were used to perform a consensus analysis of the important variables to extract potential critical quality attributes and critical process parameters predictive of T-cell potency, safety, and consistency at the early stages of the manufacturing process. Consensus analysis of the relevant variables extracted from each machine learning model was done to identify consistent predictive features of quality at the early stages of manufacturing. First importance scores for all features were measured across all ML models using *varImp* with *caret* R package except for scores for SVM which *rminer* R package was used. These importance scores were percent increase in mean squared error (MSE), relative importance through average increase in prediction error when a given predictor is permuted, permuted coefficients values, absolute coefficient values, weighted sum of absolute coefficients values, and relative importance from sensitivity analysis determined for RF, GBM, CIF, LASSO, PLSR, and SVM, respectively. Using these scores, key predictive variables were selected if their importance scores were within the 80^th^ percentile ranking for the following ML methods: RF, GBM, CIF, LASSO, PLSR, SVM while for SR variables present in >30% of the top-performing SR models from Data Modeler (R2≥ 90%, Complexity ≤ 100) were chosen to investigate consensus except for NMR media models at day 4 which were considered a combination of the top-performing results of models excluding lactate ppms, and include those variables which were in > 40% of the best performing models. Only variables with those high percentile scoring values were evaluated in terms of their logical relation (intersection across ML models) and depicted using a Venn diagram from the *venn* R package.

**Supplementary References**

1. Dwarshuis NJ, Song HW, Patel A, Kotanchek T, Roy K. Functionalized microcarriers improve T cell manufacturing by facilitating migratory memory T cell production and increasing CD4/CD8 ratio. *bioRxiv*. Published online 2019:646760.

2. Holmes E, Cloarec O, Nicholson JK. Probing Latent Biomarker Signatures and in Vivo Pathway Activity in Experimental Disease States via Statistical Total Correlation Spectroscopy (STOCSY) of Biofluids:  Application to HgCl2 Toxicity. *J Proteome Res*. 2006;5(6):1313-1320. doi:10.1021/pr050399w

*Note: These references are also used in the main manuscript but using other numbering.*
